# The Duty of the Hour

**Published:** 1867-10

**Authors:** W. E. Magill


### THE DUTY OF THE HOUR.

BY DR. W. E. MAGILL.

Read before the Lake Erie Association of Dentists.

GENTLEMEN OF THE ASSOCIATION:—The remarks to which your attention is for a short time asked, in obedience to your appointment made some months since, may be somewhat rambling, and not well-digested; but will be grouped under the general head of

"THE DUTY OF THE HOUR."

Commonly, the duty of the hour for each of us is, the work immediately before us. "*Whatsoever thy hand findeth* to do *that* do quickly," conveys to us the broad command and direction for our life-work, by one who knew that human existence is short, and intensely appreciated the value of moments. But there are special obligations, growing out of the relations which we sustain, as individuals to others, or as members of the profession which we have chosen for our life-pursuit, and these come more particularly within the scope of our present essay.

One of the military maxims credited to the elder Napoleon is this: "The army which remains within its intrenchments is already defeated;" and it sounds like an emanation from that wonderful concentration of energy and executive ability, to whose fierce determination the mighty Alps were but brief impediments, and who, in one half a short lifetime, exchanged a lieutenant's sword for the sceptre of an empire, and conquered nearly an entire continent.

In its application to affairs purely military, the author of this maxim proved its truth. No cowering behind intrenchments, no awaiting the slow approach of an enemy; but, rather, bold strategy, wonderful marches, the sudden application of new means to attain old ends, or of old means to attain new ends. The very thunderbolt of war, he hurled his legions upon the enemy with such wild impetuosity that his name became only the synonym of victory.

In politics, who is the successful man? Not the sedate conservative; not the timid vacillator; but the bold man who plans acutely, who labors hard to adjust the right wires in the right place, so that all will pull together; who promptly takes possession of the weak points in human character, and turns them to his own account.

In religion, "The blood of martyrs is the seed of the church;" and the church militant is the church triumphant. The glory of the coming millennial time is only promised when He shall have put all enemies under His feet.

The law of progress is life, energy, activity. In God's great universe *rest* is only another name for *rust*.

From this standpoint, as members of a profession whose march is upward and onward, surveying the broad field of effort before us, do we discover anything which it is clearly our duty to accomplish?

We have to preach no grand crusade to recover from the grasp of infidel hands the holy sepulchre. It is not our duty, with lance at rest, and visor closed, to charge down upon Saracen hosts, with flying pennons and the stirring battle-cry of ha! Beauseant! But all around us are the idolatrous shrines of dogmas that should be defunct; of threadbare theories and fossilized errors, only awaiting the hammer of some bold iconoclast who dares to shiver them in pieces. And far away, over the waste of waters, out in the depths of the Great Unknown, beyond the waves of experiment and toil and tribulation and weary watching, lies the golden land of Discovery, full of all things which men desire, and with silvery sands and flickering lights awaits the coming of whatever bold Columbus shall dare the stormy main.

But the profession, equipped and militant, has some errors to combat which it may be well to particularize. And first, among ourselves, is the crying wrong of *professional incompetency*, having for its foundation the common error which supposes that any man who possesses average intelligence and mechanical ability, especially if he has learned the use of watchmakers' tools, is prepared to open an office and successfully practice Dentistry. Now who is largely responsible for this state of things? Not the young man who anxiously, earnestly, but wearily, is plodding along the road *we* know so well; not *he* and *they*, but *you* and *I*, my brother, who, having passed the tribulations of a thorny road, fail to send back a warning voice and point to the better way, once shut, now free to all, thanks to those earnest men who open wide the doors of Dental Colleges. The extensive prevalence of this error may also be chargeable, to some extent, to the want of information, of a proper standard for professional ability in communities where the experiments of such beginners are made. But this fact comes home to us in the form of a question whether it is not our duty, each in his place, to sow broadcast the seeds of information among the people, that they may spring up and bear fruit, "some fifty and some an hundredfold." We must, each in his own way, become standard bearers, holding up our right royal banner inscribed "Excelsior," and rallying under its folds all whom word and example can influence.

Second in consideration, but hardly second in importance, is the appalling fact, that within the States which united (as I hope they will soon *all* again be), constitute our own loved land, an army of seven thousand men, in time of peace, is fully occupied as physicians, surgeons, blacksmiths, or undertakers, in treating, repairing, wrenching, or performing the last sad offices for American teeth! Underlying this awful fact is an error, or an accumulation of errors, which it is our duty to fight, as St. George did the dragon, and never be satisfied until every hydra head is destroyed. Americans have muscle, and brain, and nervous force; but have they in general bones of equal compactness, or teeth of equal density, compared with those of foreigners? Are their teeth as difficult to extract? If not, what are the causes, and to what extent can we use prevention and cure?

During the recent trial of the case of Goodyear against

Wait, in the city of New York, for alleged infringements of rubber patents, Thomas R. White, of New York, testified that in the year 1862, he sold 1,500 pounds of rubber for Dental purposes. In 1863, 2,800 lbs. For the year ending July 1st, 1865, 3,150 lbs. For the year ending July 1st, 1866, 4,120 lbs. It is estimated that a pound of rubber will make eighteen plates, therefore from the sales of this one Dental depot we find material furnished, in one year, for *seventy-four thousand one hundred and sixty plates!* Are not figures startling when they tell us such truths? Is this terrible "slaughter of the innocents" to go on forever, and in an increased ratio to keep pace with our so-called advance in civilization? Forbid it, Heaven! And oh, my brother sentinels, whose duty it is to guard well the living temple of human happiness, let us determine that henceforth that duty shall be well performed.

The facts that teeth, after development and eruption, retain their size, shape, color, or different shades of color, even those defects due to diseased action, or arrest of development; that during a lifetime the tooth broken in early youth remains without any restoring process from the hand of Nature, usually so prompt to repair a broken bone; these facts, I say, teach us the urgent necessity of coming in *early* to the rescue, of proving the greater benefits of *prevention* over cure, of furnishing the child with proper food; for in this matter of tooth development there is great force in the often quoted line, "The boy is father to the man," The organs of assimilation must have something to assimilate, the organs of nutrition must have nutriment to draw upon. As well order a mechanic to build a house without materials as expect Nature to construct perfect bones and teeth when the elements of such structures are carefully excluded from our food.

Parallel with these notorious facts in regard to our defective teeth, is the fact so noticeable to foreigners, that we are a people of sallow complexions, of great nervous irritability, of dyspeptic habits. That we turn with disgust from the mountain of roast beef which John Bull loves to climb, or rather *go through:* that we faint at the first odor of saur kraut, in the fumes of which Mynheer Vanderpool scents billows of blissful perfume; that we call the man a savage who takes his steak reeking and red and juicy, as the Indian epicure is sure to do.

Statistics not yet compiled must prove or disprove whatever theories may be advanced as to the causes, near or remote, of such physical peculiarities, or deviations from the normal condition of health, as we have been considering; but probably the limited observation of each one here will agree in this, that not among the people of New England, where corn meal and unbolted wheat flour are common articles of diet, nor among those of the Southern States, where "hog and hominy" compose the most popular dish in family feasts, and corn bread is held in high esteem; but rather in the middle region of buckwheat cakes and whitest of wheat flour, do we find the most defective development of teeth.

That, as a people, Americans mismanage the organs of digestion, and that their teeth suffer in consequence, must be conceded. How much we, professionally, are responsible for this condition of things, each man may consider for himself. But, unquestionably, if we throw the weight of our combined influence in favor of dietetic reform we will accomplish valuable results; and if our brethren of the medical profession join with us heartily in such a movement, the united forces will move on conquering and to conquer.

